# Venezuelan Equine Encephalitis Virus Capsid—The Clever Caper

**DOI:** 10.3390/v9100279

**Published:** 2017-09-29

**Authors:** Lindsay Lundberg, Brian Carey, Kylene Kehn-Hall

**Affiliations:** National Center for Biodefense and Infectious Diseases, School of Systems Biology, George Mason University, Manassas, VA 20110, USA; lhill10@masonlive.gmu.edu (L.L.); bcarey4@masonlive.gmu.edu (B.C.)

**Keywords:** VEEV, eastern equine encephalitis virus, western equine encephalitis virus, capsid, CRM1, importin

## Abstract

Venezuelan equine encephalitis virus (VEEV) is a New World alphavirus that is vectored by mosquitos and cycled in rodents. It can cause disease in equines and humans characterized by a febrile illness that may progress into encephalitis. Like the capsid protein of other viruses, VEEV capsid is an abundant structural protein that binds to the viral RNA and interacts with the membrane-bound glycoproteins. It also has protease activity, allowing cleavage of itself from the growing structural polypeptide during translation. However, VEEV capsid protein has additional nonstructural roles within the host cell functioning as the primary virulence factor for VEEV. VEEV capsid inhibits host transcription and blocks nuclear import in mammalian cells, at least partially due to its complexing with the host CRM1 and importin α/β1 nuclear transport proteins. VEEV capsid also shuttles between the nucleus and cytoplasm and is susceptible to inhibitors of nuclear trafficking, making it a promising antiviral target. Herein, the role of VEEV capsid in viral replication and pathogenesis will be discussed including a comparison to proteins of other alphaviruses.

## 1. VEEV Overview

Alphaviruses are important emerging mosquito-borne pathogens from the viral classification Group IV (+) ssRNA virus family *Togaviridae*. They are found globally and cause localized outbreaks as well as human epidemics. Alphaviruses can be split into two separate subgroups: old world (OW) and new world (NW). The OW alphaviruses are a loose collective of polyarthritic viruses endemic to Asia, Europe, Australia, and Africa. Representatives of this grouping that cause disease in humans include chikungunya virus (CHIKV), responsible for millions of instances of arthralgia; Ross River virus (RRV), the cause of epidemic polyarthritis; and Sindbis virus (SINV) and Semliki Forest virus (SFV), which cause polyarthritis characterized by fever and rash, though laboratory strains are typically considered avirulent with a few notable exceptions (reviewed in [1]). NW alphaviruses such as, eastern equine encephalitis virus (EEEV), western equine encephalitis virus (WEEV), or Venezuelan equine encephalitis virus (VEEV) typically cause severe encephalitic disease [2].

VEEV was discovered in 1935 after outbreaks of VEE in Columbia, Venezuela, and Trinidad. It was not isolated and grown in a lab, however, until 1938 [3,4]. Enzootic strains of VEEV consist of subtypes ID, IE, and II-VI. The enzootic strains have been isolated in Florida, Mexico, Central America, and South America. Epizootic strains consist of subtypes IAB and IC. Epizootic strains of VEEV have been responsible for every outbreak of the disease including an outbreak where over 200,000 humans were infected in Columbia during the 1960’s. Epizootic strains are found between northern Argentina and Florida, but mainly in Columbia, Venezuela, and Trinidad [5,6,7]. Heavy rainfalls typically correspond with outbreaks due to increases in the mosquito population [8,9]. Rodents are the primary reservoir host for VEEV with mosquitos from the genus *Culex* being the principle vector; however, mosquitos belonging to genera *Ochlerotatus* and *Psorphora* also vector the virus during epidemics [10].

In humans, VEEV causes moderate flu-like symptoms including fever, headache, myalgia, fatigue, nausea, and pharyngitis. In 4–14% of cases, severe neurological complications due to encephalitis such as confusion, seizures, photophobia, and coma can occur with approximately 1% of cases being fatal. Disease that progresses to encephalitis can lead to long lasting neurological deficits. Regardless of symptoms, the overall length of the disease is about one week [8,11,12,13].

Due to the ease of aerosolization and an extremely low infectious dose, VEEV was developed as a bioweapon by the United States and the Soviet Union during the Cold War [14]. Both the CDC and USDA classify VEEV as a biosafety level 3 (BSL-3) select agent pathogen and the National Institute of Allergies and Infectious Diseases classifies it as a Category B priority pathogen. There are currently no FDA approved vaccines or treatments for VEEV, however, the attenuated VEEV TC-83 strain is used to vaccinate military personnel and lab workers at risk of contracting the virus. VEEV is used in the laboratory as a model for alphavirus research, particularly in NW alphavirus research due to the ability to work with TC-83 at BSL-2.

## 2. VEEV Virion and Genome Structure

VEEV is approximately 70 nm in diameter with T = 4 icosahedral symmetry. The viral RNA is encapsidated by 240 copies of the viral capsid protein bound in the N-terminus of the protein. At the C-terminus, capsid is bound to the E2 glycoprotein. The glycoproteins E1 and E2 form a heterodimer and trimerize with other E1/E2 dimers and protrude from the viral envelope which is acquired from the host cell membrane during budding [1,15].

The genome is non-segmented consisting of 11.4 kilobases with two reading frames. The RNA has a 5′ cap and a 3′ poly-A tail and forms a stem loop structure at the 5′ end that acts as a promoter for replication [15]. The first reading frame starts near the 5′ end and encodes four nonstructural proteins (nsP1-4) which are translated as a large polyprotein named P1234. nsP1 is involved in capping the mRNA to protect the RNA from cellular nucleases [16]. nsP2 is responsible for regulating the packaging of the viral genome into infectious virions [17]. nsP3 interacts with host machinery to influence viral replication [18], while nsP4 is the RNA dependent RNA polymerase [19]. Together these proteins form the enzyme structure required for transcription and replication of the viral genome [20]. The second reading frame starts in the middle of the genome and is controlled by a 26S promoter on the minus strand RNA. This subgenomic reading frame encodes for the structural proteins including capsid and E1 and E2 envelope proteins [21]. Capsid is critical for binding to viral RNA to facilitate viral assembly. E2 is responsible for receptor binding, whereas E1 is the alphavirus fusion protein which facilitates fusion between the viral and endosomal membranes following endocytosis [15].

## 3. Alphavirus Replication Cycle

**Figure 1:**
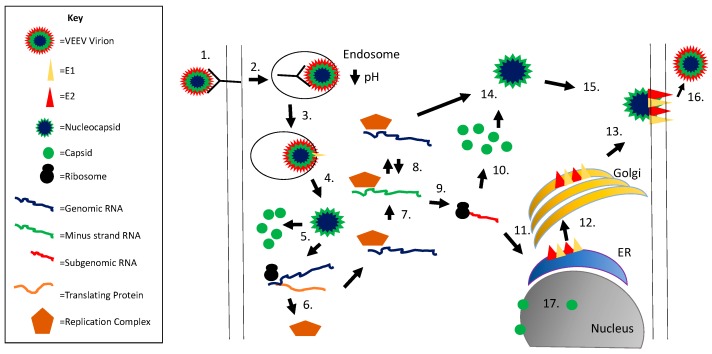
Alphavirus replication cycle. Viral entry is initiated via E2 binding to the cellular receptor (1); followed by receptor mediated endocytosis (2); the low pH within the endosome results in a conformation change in E1 and fusion of the viral and endosomal membranes (3); the nucleocapsid is released into the cytoplasm (4) followed by nucleocapsid disassembly releasing the viral genomic RNA (5); genomic RNA is translated to form the replication complex (nsP1–4) (6); which produces minus strand RNA (7); minus strand RNA serves as a template for additional genomic RNA production (8); subgenomic RNA is produced via a promoter within the minus strand RNA (9); the subgenomic RNA encodes for the structural proteins (capsid, pE2, 6K, E1); capsid is translated first (10) followed by autocleavage and translation of the remaining structural proteins on the rough ER (11); the glycoproteins are processed through the secretory pathway (12) and transported to the plasma membrane (13); capsid interacts with genomic RNA to form the nucleocapsid (14) and this complex interacts with E2 protein (15); followed by viral budding (16); capsid is also transported to the nucleus, binds importin α/β and CRM1, and blocks nuclear export (17).

Much is known about alphavirus replication (reviewed in [22,23,24]); however, less is known specifically about VEEV replication. It is generally accepted that replication across the alphavirus genus is similar, and this section will review the replication of alphaviruses as a whole (Figure 1). Alphaviruses, like most RNA viruses, replicate in the host’s cytoplasm. To initiate entry, virions attach to the host receptor through E2. E2 can bind to many different receptors including class I major histocompatibility antigen (MHC-I), α1β1 integrin, cell surface heparan sulfate, and DC-SIGN [25]. Alphaviruses are endocytosed in a clathrin-dependent manner and then transported to the early endosome. Rab5 and Rab7, markers of the early and late endosome, respectively, are necessary for VEEV entry and passage in mammalian cells [26]; the mosquito homologs play a similar role, indicating acidification is required for a productive infection [27]. The acidity of the endosome frees E1 from the glycoprotein complex, allowing for a rearrangement into a complex conducive to fusion. The hydrophobic fusion peptide of E1 is inserted in the host endosomal membrane, facilitating fusion and ultimately resulting in the creation of a fusion pore, which allow for passage of the nucleocapsid into the cytoplasm [23,28]. After release from the endosome, nucleocapsid uncoating occurs almost instantaneously. Protons flowing from the endosomal pores creates an acidic environment which facilitates disassembly of alphavirus nucleocapsid [29].

Capsid interaction with the 60S ribosomal RNA frees viral RNA for initiation of protein synthesis. The genomic RNA can be used directly for translation of the first open reading frame encoding nsP1–4. After translation, P1234 is cleaved by the nsP2 protease in cis between nsP3 and nsP4 [30] creating P123 and nsP4. P123 is then cleaved in trans by nsP2 between nsP1 and nsP2 which frees nsP1 to perform its methyltransferase activities and cap the vRNA. Finally, P23 is cleaved in trans by nsP2, freeing nsP2 to perform its RNA helicase activity [31].

RNA replication begins by polymerization of negative sense RNA which is used as a template for both genomic and subgenomic RNA by nsP4. Positive- and negative-strand RNA synthesis occurs in the cytoplasm at the membrane surface of endosomes, as has been described in SINV and SFV infections [32]. Different alphaviruses utilize specific stress granule proteins to assist in genome replication. Members of the Fragile X syndrome family are important for NW alphaviruses, including VEEV, while the G3BP family is critical for OW alphaviruses like SINV and CHIKV [33,34,35]. G3BP and Fragile X syndrome family members interact with nsP3 proteins through their hypervariable domain, assisting in the assembly of viral replication complexes [33].

The structural proteins of alphaviruses are translated as a polyprotein from the subgenomic 26S mRNA in the order of capsid, pE2, 6K, and E1. Capsid rapidly auto-cleaves itself from the growing structural polypeptide chain as the bond to be broken folds into the active site, which has been traced to the chymotrypsin-like serine protease in the C-terminal (reviewed in [1]). The autoproteolytic cleavage of capsid during translation results in exposure of the signal sequence within the new N-terminal region of the polyprotein to target the remaining structural proteins to the endoplasmic reticulum (ER) [36]. The polyprotein is then further processed through multiple cleavage events that occur within the ER or Golgi complex, resulting in the generation of E1, E2, E3, and 6K proteins (alphavirus assembly is reviewed in [1,37,38]). Signal peptidase cleaves E1 from 6K and pE2 from 6K within the ER [16,17]. pE2 is cleaved by furin protease within the Golgi complex to generate E3 and E2 proteins. E3 binds the E1/E2 spike complex and protects it from the low pH of the secretory pathway, preventing premature fusion during biogenesis [39]. Transport of the glycoprotein heterodimers to the plasma membrane occurs via cytopathic vacuoles type II (CPV-II) [40]. Newly transcribed RNA associates with capsid and assembles at the plasma membrane with the glycoproteins. The binding of capsid to E2 provides the energy needed to bud out of the cell [15].

## 4. VEEV Capsid Structure and Function

Much of what is known about the physical attributes of the alphavirus capsid protein, and VEEV in particular, was gleaned from early work with SINV and SFV. The assembled SINV nucleocapsid is approximately 400 Å in diameter and composed of 240 copies of capsid organized in a T = 4 icosahedral pattern [41]. The VEEV nucleocapsid has been confirmed to assemble in a similar manner. However, the capsomere orientation differs from other alphaviruses; the E1 glycoprotein and the C-terminal of capsid is highly conserved, so both likely adopt similar structures across the family, whereas E2 and the capsid N-terminal have limited conservation and may adopt different structures. The pentameric and hexameric capsomeres themselves are rotated counterclockwise ~11° and ~4° relative to SINV [42].

**Figure 2:**
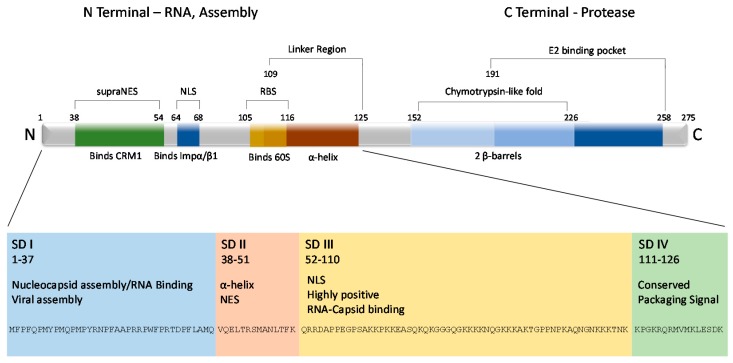
VEEV capsid protein structural layout. The VEEV capsid protein is roughly divided into two terminals, amino (N) and carboxy (C). From there, the N terminal can be further subdivided into four arbitrary subdomains (SD) based on physical structure and function. The C terminal contains the chymotrypsin-life fold, which is responsible for cleaving capsid from the growing structural polyprotein, and a binding pocket that interacts with the viral glycoproteins, contributing to the structure of the virion. The first 126 amino acids are also displayed at the bottom of the figure.

The VEEV capsid protein is 275 residues (though the capsids of EVE, MENA, 78V-3531, and AG80-663 strains of VEEV vary from 274 to 279 residues) and is divided into two distinct domains—N-terminal and C-terminal [43] (Figure 2). A highly conserved ‘linker region’ from residues 109–125 [24] connects the two domains. The linker region is arranged in a short α-helix and resides within the inner surface of the nucleocapsid core, indicating that once the virus is released to the cytosol, capsid undergoes conformational changes that expose the region to host factors [44]. Partially overlapping with the linker region is a short sequence that serves as the ribosome binding site (RBS) and binds the cellular 60S ribosomal subunit, which aids in nucleocapsid disassembly upon release in the cytosol. Originally identified in SINV, the RBS corresponds to capsid residues 105 to 116 in VEEV [45]. This sequence is also highly conserved across other alphaviruses (including WEEV, EEEV, SFV, and SINV) [46]. A deletion mutant from residues 97 to 106 in the SINV capsid did not affect assembly or particle formation, but 26S subgenomic viral RNA became incorporated into viral particles, indicating the region also plays a role in encapsidation of genomic RNA [47].

Capsid’s N-terminal domain is disordered among alphaviruses [48,49] and difficult to crystallize [50]. Thirty positively charged amino acids, lysine and arginine, and one negative residue, glutamic acid, in addition to twenty proline residues, render this domain highly disordered. Its general lack of structure suggests it protrudes into the interior of the nucleocapsid, enhancing the electrostatic interactions with the negatively charged viral RNA (reviewed in [1]). Most likely these thirty residues, which are largely hydrophobic, cluster and initiate core assembly by forming a scaffold structure [51]. A helix was hypothesized in residues 34–51 and theorized to aid in core stabilization by encouraging neighboring capsids to interact through a coiled-coil structure [52]. However, recent cryo-EM and modeling do not support the existence of such a structure [44]. Regardless, the first fifty residues contain positively charged amino acids that are likely responsible for a majority of the genomic RNA interactions [51,53], possibly through charge neutralization [47,54].

Capsid’s N-terminal region can be further divided into four subdomains, referred to as SD1–4, that are critical for nucleocapsid formation [55]. SD1 (aa. 1–37), which is only composed of 37 amino acids, is indispensable for release of both infectious virus and virus-like particles [53]. While it has very few positively charged amino acids, no predicted secondary structure, and is diverse among alphavirus species, VEEV SD1 is critical for nucleocapsid assembly and viral assembly. Its deletion or substitution of a similar sequence from other alphaviruses has a deleterious effect on infectious virus release [55]. SD2 (aa. 38–51) contains an α-helix, and in VEEV, works in synergy with SD1 to drive nucleocapsid formation through forming a central core and determining viral genomic RNA interactions [47,52,55]. Its deletion leads to nonspecific encapsidation of other RNAs [52]. SD2 also promotes the dimerization of SINV capsid [52,56]. The most positively charged subdomain, SD3 (aa. 52–110), inhibits nucleocapsid assembly until RNA interactions neutralize its charge [53]. SD4 (aa. 111–126), which is also positively charged, further mediates interaction with the viral genome and contributes to core stability. Mutations corresponding to the SD4 region contribute to nonspecific nucleocapsid formation in SINV and SFV [47,52].

Capsid’s N-terminal domain also has important implications in cytopathogenicity. Early work demonstrated that the capsid of NW alphaviruses was responsible for the host transcriptional shutoff which leads to the cytopathic effects characteristic of an alphavirus infection, similar to the nsP2 of OW alphaviruses [57], which initiates the same phenotype. Further, transcription inhibition was mapped to the N-terminal of capsid and was independent of its protease activity or the RNA binding domain [58]. Transcriptional shutdown was initially traced to a thirty-five amino acid stretch in the N-terminal of capsid, specifically residues 33–68 [59] and in later work to residues 64–68 [60]. Two important domains were described: an α-helix important in maintaining balance between nuclear and cytoplasmic localization of capsid, and a downstream peptide now known to contain a nuclear localization signal (NLS). Both regions were found to be necessary for capsid distribution throughout the cell, as well as transcriptional inhibition. Swapping out residues 33–68 with the corresponding SNV sequence attenuated the virus but did not affect replication [61]. The described peptide was also found to interfere with host receptor mediated nuclear trafficking but not passive diffusion, in mammalian but not mosquito cells [59]. Nuclear export and localization signals (NES and NLS, respectively) were eventually described in this region (Figure 2). Capsid is now known to complex with the host export karyopherin CRM1 and the import karyopherins importin α/β1 (Figure 1). It is unusual for cargo to bind to both import and export receptors due to the RanGTP gradient [62,63,64]. Based on this, a model was suggested whereby the tetrameric complex blocks the nuclear pore channel and inhibits nuclear trafficking, leading to inhibition of host transcription. Mutations in the NES and NLS ablated capsid-specific nuclear traffic inhibition [62]. Additionally, naturally occurring attenuated strains of VEEV contain mutations in this region and the inability to inhibit nuclear import [61,62,65,66].

Capsid’s C-terminal domain is highly conserved among alphaviruses and orders into crystals [48,49]. Two hydrophobic pockets have been noted in the C-terminal, though the exact structures seems to vary somewhat between alphaviruses [50,67]. Early work with SINV and SFV demonstrated one pocket contains a chymotrypsin-like fold with two β-barrel subdomains [50,67] centered around catalytic residues His141, Asp163, and Ser215 [50,68,69]. The protease sequence is conserved among chymotrypsin-like serine proteases, and all alphaviruses contain the catalytic serine (as discussed in [67]). In VEEV, the catalytic triad corresponds to His152, Asp174, and Ser226 [70]. The other pocket (corresponding to amino acids Tyr184-Trp251 in SFV and Tyr191-Trp258 in VEEV) is involved in viral glycoprotein interactions [67,71,72] and is therefore critical for viral assembly.

## 5. Capsid’s Role in Viral Assembly

Capsid’s ability to form oligomers and interact with the viral RNA and the E2 glycoprotein are all critical steps to ensure formation of infectious viral particles. Capsid associates with genomic RNA and assembles in distinct, stable structures within the cytoplasm. Capsid binds RNA in the nsP1 portion of the genome between nucleotides 856–1150 [73]. This area is known as the RNA packaging signal. Once capsid binds the RNA packaging signal, additional capsid proteins are recruited through C-terminal protein interactions with the growing structure and electrostatic interactions between the RNA and the charged N-terminal of the capsid. Early work with SFV and SINV contributed largely to what is known about these interactions in other alphaviruses. Cryo-electron microscopy coupled with atomic modeling revealed that as capsid protein joins the nucleocapsid, it undergoes a rearrangement, and a cross-linking N-terminal arm forms that fits into a cleft of the next monomer. Contact between the N-terminal and RNA then likely occurs below the adjacent subunit [72]. It is thought that these connections are highly disordered. Additionally, stretches of this region are predicted to be helical and involved in RNA packaging [74]. The RNA binding region lies between amino acids 76 and 116 of the SINV capsid [75] (Figure 2). A stretch of amino acids corresponding to residues 97 to 106 of the SINV capsid is highly conserved among alphaviruses—including VEEV, EEEV, and WEEV—and it has been shown that capsid from one alphavirus can bind to the RNA of another, in the case of SINV and SFV, indicating this sequence is indispensable for RNA binding [75,76]. Initial research with SINV revealed the capsid’s C-terminal region interacts with the cytoplasmic tails, or the C-terminal domain, of the E2 residues in the lipid bilayer [77]; it is the glycoprotein interactions that then stabilize the viral structure through formation of a scaffolding lattice that can be maintained independent of capsid [78]. This was further confirmed in greater resolution with SFV; the E2 tail binds a cleft in the C-terminal domain of capsid [72].

While particle budding occurs at the plasma membrane and the viral components are translated in the host’s cytoplasm, not much is known about the trafficking and assembly of said components. Live cell imaging of fluorescently labeled SINV capsid showed localization in distinct foci. Some capsid appeared highly mobile and localized with E2 at the plasma membrane, likely ferried by transport machinery. Membrane associated capsid recruited additional capsid, representing sites of viral assembly and egress. Nonmotile capsid colocalized with RNA stress granules or viral nsP3 [79], which are now known to be sites of viral replication complexes [33].

After assembly, the completed virion itself appears to shrink as part of a maturation event triggered through capsid—E2 interactions. Electron cryomicroscopy has confirmed that the diameter of isolated nucleocapsid is larger than that of nucleocapsid within the virion, indicating extensive conformational changes during maturation. The energy needed for such nucleocapsid reorganization likely comes from the lipid bilayer and transmembrane proteins [80,81]. Mature virions bud from the host plasma membrane. Budding is driven by glycoprotein-capsid interactions, and the viral genome is not necessary to form enveloped virus-like particles (alphavirus budding is reviewed in [38,82,83]). The glycoprotein clusters preclude host proteins; the cytoplasmic tail arrays of E2 bind cooperatively to preformed nucleocapsid, encouraging egress from the host cell [72].

## 6. Capsid’s Role in Innate Immune Response Suppression

Alphaviruses suppress innate immune responses at least partially through the inhibition of macromolecular synthesis [58,61,84,85,86,87]. Host transcription shutoff is capsid dependent in EEEV [58] and VEEV [58] infections, whereas in OW alphaviruses nsP2 is responsible [58,84] (Table 1). The capsid protein of the NW alphaviruses, that is EEEV, VEEV, and WEEV, are strongly conserved across both the C- and N-terminals [1,88,89]. The ability of VEEV capsid to shutdown transcription has been largely attributed to its ability to block nucleocytoplasmic trafficking. VEEV has an NLS and supraNES, enabling it to shuttle between the host’s nucleus and cytoplasm [59,62]. A supraNES is an NES that is able to interact with CRM1 in the absence of RanGTP. Knocking down or chemically inhibiting the host import and export proteins (karyopherins) that interact with VEEV capsid alters its localization patterns and reduces viral titer [90]. The NLS and NES are conserved across WEEV and EEEV capsid sequences [62], and chemically targeting host karyopherins reduces titers of both viruses [91], suggesting that nucleocytoplasmic trafficking of capsid occurs with those viruses as well.

The EEEV capsid, which is also known to traverse the nucleus [92], has been shown to inhibit gene expression of reporter genes under the control of RNA polymerase II promoters, but not T7 RNA polymerase promoters. EEEV infected cells display decreased cellular mRNA accumulation, phosphorylation of eukaryotic initiation factor 2 alpha (eIF2α), and an overall inhibition of host protein synthesis [85]. These events were shown to be EEEV capsid dependent. A five amino acid deletion of the EEEV capsid, mapped to residues 65–69, which correspond to a putative NLS, attenuated viral replication in mammalian cells, but not mosquito cells, and renders the virus more sensitive to interferon [85,92]. These results indicate this region is critical to inhibiting host gene expression and protecting the virus from the interferon-mediated antiviral response.

WEEV is a naturally-occurring recombinant virus derived from EEEV and a SINV-like virus. Most of the genome is similar to that of EEEV, including capsid, but its glycoproteins were derived from the SINV genome [1,99]. The recombination events likely occurred over one thousand years ago, based on the currently circulating EEEV strain and its divergence from the common ancestor of WEEV [99]. The arrangement of the WEEV nucleocapsid is more similar to OW alphaviruses, specifically SINV, than other NW alphaviruses; the capsomere orientation differs compared to VEEV even though the sequence is most similar to EEEV [100]. WEEV capsid has been found to inhibit pattern recognition receptor pathways [94]. Although the exact mechanism for this inhibition is unknown, this inhibitory activity was mapped to downstream of activated interferon regulatory factor 3 (IRF-3), a mediator of the cell-intrinsic innate immune response that is triggered by WEEV infection [94]. To date, no studies have shown the ability of WEEV capsid to shut down host macromolecular synthesis. As WEEV’s capsid protein is derived from EEEV and its nsP2 protein from SINV, it would be interesting to determine the contribution of each to the inhibition of host transcription.

There are indications that OW alphavirus capsid proteins may have roles beyond binding to RNA and serving as a protease. An NLS was found in the CHIKV capsid that interacted with both importin α and karyopherin 4, resulting in CHIKV capsid nuclear translocation. In addition, CHIKV capsid was shown to contain a functional NES, facilitating the interaction with CRM1, and resulting in CHIKV capsid nuclear export [101]. As these findings were demonstrated in a virus-free system, it has yet to be determined if CHIKV capsid shuttles in and out of the nucleus during a viral infection. Chemically inhibiting CRM1 reduces the infectious titer of CHIKV, though not to the extent seen with NW alphaviruses, and has no affect at all on SINV titer [91]. SFV capsid contains two nucleolar targeting signals (NOS), likely attributing to its karyophilic nature [102]. Bioinformatic analysis identified human host proteins predicted to interact with CHIKV capsid and the other structural proteins [103]. Processes involving signaling cascade activation, apoptosis, and positive regulation of the immune response were enriched in this analysis [103]. Much work remains to be done when it comes to identifying and describing the host interactions that occur with capsid of OW alphaviruses, as a vast majority of research has focused on nsP2 as the inhibitor of host transcription.

## 7. nsP2 Inhibits Innate Immune Responses

OW alphaviruses also shut down host transcription and dampen the immune response [84], but unlike NW alphaviruses that utilize capsid, these affects are attributed to the nsP2 viral protein (Table 1) (reviewed in [57]). nsP2 has both NTPase [104] and helicase activity [105]. In the virus lifecycle, nsP2 acts as the protease that cleaves the nonstructural polyprotein [106]. Additionally, nsP2 has a role in the initiation of 26S mRNA synthesis [107] and the switch from early to late phase replication as well as manipulating the host’s response to infection [108] (reviewed in [1,109]). Through immunofluorescence and cellular fractionation, it was found that SFV nsP2 was the only nonstructural protein that transported to the nucleus and associated with the nucleoli [110]. An NLS and nucleolar targeting sequence was identified but not required for virus replication [109,111]. However, if the sequence was mutated, the virus was still neuroinvasive, but its pathogenicity was attenuated in adult mice infected intraperitoneally. Lethality was restored if the mutated virus infected interferon α/β receptor deficient mice [112,113]. In contrast, while initial reports indicated that VEEV nsP2 localizes to the nucleus [114], later studies suggest that VEEV nsP2 does not translocate to the nucleus, and its presence in the nucleus at low concentrations can be traced to capsid’s disruption of nucleocytoplasmic trafficking [59]. Further defining the interactions between host cells and the nsP2 of OW alphaviruses, it was found that SINV nsP2 induced cytopathic effect and cell death, shut down host transcription, and that these functions were independent of its helicase and protease functions [84]. SINV nsP2 also decreases interferon production; mutations make the virus less cytopathic and allow persistence in cell lines with defective interferon α/β signaling [115]. Through its helicase and *S*-adenosylmethionine-dependent methyltransferase-like domains, SINV nsP2 mediates ubiquitination of host Rpb1, a component of the RNAPII complex. This leads to downregulation of the cellular antiviral response at the transcriptional level [95]. CHIKV nsP2 has been shown to inhibit the phosphorylation of STAT1 and block its nuclear translocation, leading to a dampening of interferon induced JAK-STAT signaling that shuts down the host innate immune response [96]. Additional host proteins that interact with the nsP2 of CHIKV were identified using a high-throughput yeast two-hybrid assay and include proteins involved in translational machinery, RNA splicing factors, and cytoskeletal components. Many of these interactions were also confirmed with nsP2 from SFV and SINV [116] and corroborated previous findings [117,118,119].

VEEV nsP2 is capable of blocking host macromolecular synthesis and suppressing innate immune responses in the absence of capsid expression. Specifically, inhibition of STAT1 nuclear translocation and subsequent interferon-α and -γ signaling was observed in cells exposed to VEEV replicon particles, indicating that one of the nsPs are responsible for this affect [93]. Similarly, inhibition of cellular translation occurred in cells infected with VEEV replicons [86]. Recent results present conclusive evidence that VEEV nsP2 is responsible for host translational shutoff, where expression of nsP2 alone, but not nsP2 mutant Q739L, resulted in decreased protein synthesis [87]. VEEV mutated at this site, VEEV nsP2 739L, exhibited decreased inhibition of host macromolecular synthesis and attenuation of pathogenesis in mice. Expression of EEEV nsP2 did not result in shutdown of host translation [87], highlighting a significant different between these two NW alphaviruses. Given the temporal differences in viral gene expression, with nsP2 being produced immediately after entry, nsP2 is likely to be important for the resistance of VEEV to antiviral responses.

## 8. Capsid as a Target for Therapeutic and Rational Vaccine Design

TC-83 is a live attenuated strain of VEEV that has been used as a vaccine in equines and humans. It was generated by passaging the virulent Trinidad Donkey (TrD) strain 83 times in guinea pig heart cells. TC-83 has limited immunogenicity, with only about 80% of individuals seroconverting and is fairly reactogenic (40% in vaccinated individuals) [120], with individuals developing flu-like symptoms [21]. There is also concern that it may revert to the wildtype virulent form due to its attenuation relying on only two point mutations, one in the E2 glycoprotein and the other in the 5′UTR [121]. Based on TC-83’s limited utility, alternative VEEV vaccine strategies are being explored, one of which involves further attenuating TC-83 through mutation of capsid. This strategy is based on data indicating that mutation of VEEV capsid in the N-terminal NLS region (amino acids 64–68) decreases the ability of the virus to induce cytopathic effects, without effecting viral replication [122]. Capsid mutant virus (VEEV/Cm) caused persistent infection of cells and allowed innate immune response (e.g., interferon productive) activation [60,122]. Infection of suckling mice with VEEV/Cm resulted in viremia, neuroinvasion and lethality in a small percentage of mice; whereas infection with TC-83 results in 100% mortality in these mice [60]. Further attenuation of VEEV/Cm through targeting VEEV’s packaging signal (VEEV/PS−/Cm) or putting capsid expression under the control of the internal ribosomal entry site (IRES) of encephalomyocarditis virus (EMCV) (VEEV/IRES-Cm), resulted in further attenuation with no mice succumbing to infection [60]. Vaccination of 6-week old mice with all three of these attenuated viruses resulted in high concentrations of neutralizing antibodies and complete protection against challenge with the virulent epizootic VEEV strain 3908 [60]. These studies indicate that disrupting capsid’s ability to alter nucleocytoplasmic trafficking significantly attenuates VEEV and is a viable strategy for developing a safe and effective live attenuated vaccine.

The ability to inhibit capsid’s functions has also attracted attention as a potential therapeutic target. Many successful antiviral drugs target viral enzymes, including RNA polymerases and proteases. Capsid’s protease site becomes inactive after cis cleavage of capsid from the polyprotein due to a C-terminal tryptophan that sits in the P_1_ substrate site, blocking it from further activity [50]. Therefore, development of a drug to target capsid’s protease activity is unlikely. In contrast, efforts to inhibit VEEV capsid’s ability to interact with importin α/β and CRM1 have been successful. Nuclear import inhibitors mifepristone and ivermectin [123] block VEEV capsid nuclear import, decrease VEEV replication, and limit VEEV induced cytopathic effects [90]. Likewise, inhibition of CRM1 through selective inhibitors of nuclear import (SINE) compounds significantly decrease VEEV replication through limiting the pool of capsid available for viral assembly via preventing capsid export from the nucleus [91]. SINE compounds also inhibited EEEV and WEEV replication, suggesting this strategy may be useful as a pan-encephalitic alphavirus therapeutic [91].

## 9. Future Perspectives

The study of VEEV capsid as it relates to viral replication and inhibition of the host response have intensified in recent years. However, there are additional aspects of capsid biology that are yet to be explored. One such aspect is the importance of capsid binding to viral RNA to influence events independent of viral assembly. Recent work with SINV has shown that capsid binds to additional sites on the viral RNA outside of the packaging signal [124]. Mutation of these sites resulted in decrease stability of incoming viral RNA and reduced virulence in mice. These results suggest that SINV capsid binding to viral RNA may also assist in stabilizing viral RNA early in infection. Expansion of this work to additional alphaviruses, including VEEV, would be of great interest to the alphavirus community.

A second area in need of increase research focus is characterization of additional capsid:host protein interactions. Currently, capsid’s ability to inhibit host transcription is largely attributed to it forming a tetrameric complex with CRM1 and importin α/β proteins, resulting in obstruction of the nuclear pore [62]. However, there are likely to be additional host protein interactions that contribute to the ability of capsid to inhibit host transcription. Likewise, the EEEV and WEEV capsid protein interactome is unknown. A proteomic analysis of capsid:host protein interactions should shed light on additional roles of capsid as well as provide novel targets for rational therapeutic design.

Another area of interest is the characterization of capsid post-translational modifications. Protein post-translational modification is a commonly used mechanism to regulate protein function. However, to date, little work has been done to characterize capsid post-translational modifications. VEEV capsid was shown to be both mono- and poly-ubiquitinated early during infection (within 6 h) as well as within virions [125]. The poly-ubiquitin chains were formed through K48 linkages [125], which are often associated with targeting proteins to the proteasome for degradation [126]. Importantly, inhibition of the ubiquitin-proteasome system, through bortezomib treatment, resulted in decreased VEEV replication, suggesting that capsid ubiquitination is important for viral replication. To date, no other post-translation modifications of capsid have been reported, although preliminary work in our lab has detected capsid phosphorylation. Given the well-known role of post-translation modifications in viral capsid assembly and regulation [127,128,129,130], this is an area of research that is in need of further investigation.

## Figures and Tables

**Table 1:** Innate Immune Evasion Strategies Employed by Alphavirus Proteins.

Virus	Viral Protein	Pathway/Responses Modulated	Host Protein Affected ^a^	References
VEEV	capsid	Transcription, Nucleocytoplasmic trafficking	CRM1, Importin α/β1	[58,59,61,62]
VEEV	nsPs	Interferon beta and gamma signaling	STAT1	[93]
VEEV	nsP2	translation	Unknown	[87]
EEEV	capsid	Transcription, Translation	eIF2α	[85,92]
WEEV	capsid	Pattern recognition receptor pathways	Unknown	[94]
SINV, SFV, CHIKV	nsP2	Transcription	Rpb1	[95]
CHIKV, SINV	nsP2	Jak/STAT and interferon signaling	STAT1/2	[96,97]
CHIKV	nsP2	Translation, Transcription, Unfolded Protein Response	eIF2α	[57,98]

^a^ Proteins may be directly or indirectly modulated by the viral protein noted.
